# Longer healthy life, but for how many? A stochastic analysis of healthy lifespan inequality

**DOI:** 10.1007/s10479-024-06203-1

**Published:** 2024-08-12

**Authors:** Virginia Zarulli, Hal Caswell

**Affiliations:** 1 Department of Statistics University of Padova, Padova, Italy; 2 Faculty of Science, Institute for Biodiversity and Ecosystem Dynamics University of Amsterdam, Amsterdam, The Netherlands

**Keywords:** Healthy life expectancy, Health adjusted life expectancy, Healthy life expectancy variation, Global burden of disease, Markov chain model, Markov chains with rewards

## Abstract

Over the past 150 years, life expectancy doubled and healthy life expectancy increased. Expectations reveal nothing about variability, so we present a stochastic analysis to investigate changes over time, age and gender of variation, among individuals, in healthy lifespan, for different levels of country income. To complement health-adjusted life expectancy (HALE) data from the Global Burden of Disease Study, we use a stochastic model to compute the standard deviation of healthy life (SDHL). The model is a finite-state absorbing Markov chain with rewards. It includes stochastic survival, mortality, and loss of good health status. An individual surviving from one age to the next gains, as a "reward," a year of good health. This method provides all the moments of healthy longevity. The mean healthy longevity is exactly the HALE. As a measure of variation, here we focus on the standard deviation of healthy longevity. From 1990 to 2019, HALE increased, with greater increases at younger ages. At the same time, SDHL at younger ages decreased and at older ages increased. The most significant changes at birth occurred in low- and lower-middle-income countries. High- and upper-middle-income countries saw notable increases at old ages. Women generally have longer HALE and higher SDHL, but the overall HALE increase was greater for men. The reduction in SDHL over time suggests that more individuals benefit from increased longevity, particularly in low-income countries closing the gap with high-income countries. However, improvements in healthy survival at older ages appear unevenly distributed among individuals in high-income countries.

## Introduction

Differences in how many years in good health individuals can expect to live are a fundamental inequality of population health. Some have claimed that, because every other inequality depends on being alive or not, the most fundamental inequality of all is thus the inequality in the length of life (Van Raalte et al., 2018), although inequality in length of life differs in important ways from other kinds of inequality (Caswell, 2023). But being alive and healthy is as crucial as being alive, perhaps even more crucial, as most of the relevant dimensions of social and population inequality we can think of, from gender to socioeconomic status, are much more substantiated and affect more the life of the individuals when these are alive and in good health than when they are simply alive. Surviving in good health is the essential basis for acquiring human capital through education, training, experience and social mobility. Healthy years of life are also a crucial part of the dynamics of the life cycle: as long as individuals are healthy they are more likely to be productive, thus being net contributors to the society, as showed by the stronger correlation of the expected number of working years with healthy life expectancy than with (total) life expectancy (Loichinger & Weber, 2016).

As with any average quantity, similar levels of healthy life expectancy can be achieved by different distributions of healthy lifespans: some more concentrated, where many individuals share more similar number of years in good health, and others less concentrated, where some individuals enjoy substantially higher numbers of years in good health than other individuals, who suffer from ill health early on. How equally (or unequally) the healthy years of life are distributed between the individuals is one of the fundamental distributional problems of population health. Studying patterns and trends of the distribution of healthy lifespans among the individuals is a crucial dimension of populations health, because a healthy population is one in which not only people live a long healthy life on average, but also where long healthy lives are enjoyed by everybody. Ensuring longer and healthier lives for as many individuals as possible has become a pressing, international priority, with the mandate of Goal 3 of the Sustainable Development Agenda of the United Nations. The study of healthy life space variation is relevant also for its crucial socioeconomic implication, for example for retirement and health policies, as highlighted by the analysis of the trends in working life expectancy at age 50 in Europe, which found that this indicator has a higher correlation with healthy life expectancy (based on self-reported health) than with total life expectancy (Loichinger & Weber, 2016). Finally, shedding light on the patterns of healthy lifespan variation is also relevant for the so called male–female survival paradox, which is the fact that women live longer but have poorer health than men (Ahrenfeldt et al., 2019; Oksuzyan et al., 2008). Recent results suggest that this paradox is a function of indicators of population health and their gender differences Van Oyen et al. (2013). Undoubtedly, healthy lifespan variation is such an indicator and, yet, indicators of healthy lifespan variation are missing from the landscape of gender differences in health demography.

Since the concept of healthy life expectancy was introduced by Sanders (1964) and later operationalized by Sullivan (1971), the increasing interest in healthy longevity has generated a vast literature (e.g., Murray et al., 2000; Robine et al., 1999; Siegel & Olshansky, 2012; Salomon et al., 2012) that has focused, however, on expected values and inequalities between groups, disregarding the fundamental distributional question of how the healthy lifespan is distributed between the individuals within the populations. In the last decade or so, researchers working on mortality have recognized the importance of supplementing analyses of average longevity (such as life expectancy) with analyses of variation in lifespan length in the assessment of population health (Van Raalte et al., 2018), producing a wealth of research (e.g., (Van Raalte et al., 2018; Aburto et al., 2020; Edwards & Tuljapurkar, 2005; Edwards, 2011; Engelman et al., 2010; Permanyer et al., 2018; Sasson, 2016; Smits & Monden, 2009; Van Raalte and Caswell, 2013; Van Raalte et al., 2011; van Raalte et al., 2012, 2014; Vaupel et al., 2011; Wilmoth and Horiuchi, 1999; Caswell, 2023)) whose main message is that, as life expectancy has increased, the ages at death have become more similar, even though with some exceptions and differences by gender and social class.

What about the healthy years of life? We know that today people reach older ages in better health than what they used to do only a few decades ago because there has been a general improvement of healthy survival and a postponement of debility (Ahrenfeldt et al., 2018; Christensen et al., 2009; Jeune & Brønnum-Hansen, 2008; Vaupel, 2010). But we don’t know what has happened to the distribution of this longer healthy lifespan among individuals: do more individuals enjoy increasingly similar number of years of healthy life, or the distribution has become more unequal?

Some insights come from the literature on the compression of morbidity, which shows a shift to older age of the entry into the morbid (unhealthy) state. These results seem robust despite the numerous differences in the definitions of disability, morbidity and functioning indicators used in the analyses (Mor, 2005; Chatterji et al., 2015), even though there are notable exceptions such as the United States, where an inversion of trend has been reported between 1998 and 2006 (Crimmins & Beltrán-Sánchez, 2011). However, the analysis of the compression of morbidity does not equal the analysis of variation in healthy lifespan and is not necessarily informative about the distribution of healthy lifespans within the populations because this is determined by the interaction between the structure of morbidity and of the mortality schedule.

The growing availability of data on health and mortality has naturally led to an increased interest in the theoretical issues related to the topic of healthy lifespan inequality (Permanyer et al., 2022) and to studies of variability of healthy years of life in Europe (Muszyńska-Spielauer et al., 2024; Permanyer et al., 2022) and on a global scale (Zarulli and Caswell, 2022; Permanyer et al., 2023). To our knowledge, so far only one study other than the present one has explored healthy lifespan variation and its implications at the global level (Permanyer et al., 2023). Using the metric of HALE (Health-Adjusted Life Expectancy), those authors found that globally, between 1990 and 2019 variation on healthy lifespan at age 65 increased for both sexes, emphasizing that while improvements in longevity were evident, they did not necessarily correspond with reductions in healthy lifespan variation. Furthermore, while morbidity tended to compress overall, particularly in low and middle-income countries, it stagnated in high-income nations. Notably, the variability in the ages at morbidity onset was found to be larger than the variability in lifespans, a divergence that widened over time.

Their results remain the only ones so far and await confirmation by further analyses. In this study, we use the same dataset of Health-Adjusted Life Expectancy but employ different methods. Unlike previous studies, we go beyond life table methods and use an explicit stochastic model for healthy longevity. The resulting model has many advantages over life table methods: it can be extended to many different kinds of health states, it provides as many moments of healthy longevity as desired, and can be extended to multistate life histories.

Health adjusted life expectancy (HALE) is a measure of average longevity that combines both mortality and morbidity. It is the average number of years that a person, of a specified age, can expect to live in "full health," where health can be defined according to the interests of the investigator. Years of healthy longevity can be lost to mortality and to years lived in less than full health due to disease or injury. HALE is used by numerous international organizations, including the World Health Organization (WHO). It is calculated from the data of the Global Burden of Disease (GBD) Study (Institute for Health, 2019; Lopez & Murray, 1998). We use this measure to explore the variation among individuals in the length of healthy life at the global level, using the estimates provided for 204 countries by the GBD. This systematic assessment of the trends in healthy lifespan variation provides a global picture of the international landscape of inequality in healthy longevity among individuals.

The variance among individuals in longevity or healthy longevity calculated from any life table or Markov chain is the result of stochasticity, not of heterogeneity (or, in a strict sense, inequality) among the individuals (Caswell, 2023). This is because the calculations explicitly treat every individual of a given age as subject to the same demographic rates. Differences among individuals in the outcome are the result of chance. Even so, this variation is real and has social and economic consequences. It is also important to note that this variation arises from demographic process and does not represent sampling or estimation errors.

We hypothesize that, as healthy life expectancy has increased, healthy lifespan variation has decreased, following the same negative relation found between life expectancy and lifespan variation. However, the relation between healthy lifespan length and healthy lifespan variation may differ among ages because health systems might be more successful at preventing health deterioration at some ages than at others, due to various factors ranging from available medical technology to societal preferences towards preventing health deterioration of specific age groups (for example children and young adult rather than old ages). Furthermore, the variation in healthy years of life lived depends on the interaction between the age-specific structures of health prevalence and survival, which may change differently over time. Finally, historically, we know that the first to benefit from reductions in the uncertainty of death were the young ages, which were gradually followed by the older ones (Vaupel et al., 2011). Therefore, it is reasonable to think that the evolution over time of healthy lifespan inequality and its relation with the healthy lifespan length can differ by age.

We also expect to find differences among countries in the level of healthy life span variation and its relation with the healthy lifespan length. Such differences could be related to the socio-economic level of the countries. Again, we derive this hypothesis from past and present knowledge. First, the fact that the leading countries in terms of life expectancy increase were the ones who first succeeded in reducing individual variation in the age at death (Vaupel et al., 2011), and the various phases of the epidemiological shift proceeded in cycles of divergence between countries, followed by convergence, when the late comers caught up with the forerunners (Meslé & Vallin, 2011). Country differences in the level of development, organization of the welfare systems, education levels and the general life standards have played a role in this differentiation. Second, the fact that today there are still considerable differences in welfare systems (Esping-Andersen, 1990), allocation of the health expenditures (Glied & Smith, 2013) and socioeconomic indicators. These are not only associated with both health and mortality outcomes (Kim & Kim, 2016) and greatly depend on the level of wealth of the country, but also partly reflect the value a society assigns to health. Therefore, it i reasonable to expect that healthy life span variation may differ by the country socioeconomic level, in ways that still need to be investigated.

Finally, we anticipate to find higher healthy lifespan variability in women compared to men, contrary to what happens for the variation in total lifespan. Women live longer than men (Austad & Fischer, 2016; UN, 2023) and have less variable total lifespans than men (Permanyer et al., 2018; Sasson, 2016; Colchero et al., 2016). The male–female health-survival paradox tells us that men are physically stronger, have fewer disabilities and report higher health scores, while showing substantially higher mortality at all ages compared with women (Oksuzyan et al., 2008; Crimmins et al., 2011; Oksuzyan et al., 2014; Alberts et al., 2014; Ahrenfeldt et al., 2019). This results in women having, yes, a longer healthy life expectancy than men, but a smaller health advantage over men than what is observed for total life expectancy. This could be due, at least partly, to women having higher healthy lifespan variation than men because such a wider distribution means higher numbers of women losing their health at ages below the average value (healthy life expectancy) compared to men.

With this analysis we intend to delve into these questions, each serving as a compelling research inquiry warranting thorough exploration in future analyses that we plan to conduct.

## The Markov chain model for healthy longevity

Variation among individuals in healthy longevity results from two stochastic processes. One is the continual chain of risks to which an individual is subject; living to a specified age requires having the good fortune to avoid the accumulated risks of mortality up to that age. The second process is the chance of encountering a disabling condition, estimated by the prevalence of that condition among individuals at each age. Our model, known as a Markov chain with rewards, accounts for both these processes. A Markov chain describes the stochastic transitions of individuals from one age class to the next. A ‘reward’ process incorporates the chance outcome of the disabling process. The model calculates the mean, the variance and all other moments of lifetime accumulated reward; that is, the remaining healthy longevity from each age.

The life of an individual is described by an absorbing Markov chain with transition matrix

1 $$\begin{aligned} \textbf{P} = \left( \begin{array}{c|c} \textbf{U} &{} \boldsymbol{0} \\ \hline \textbf{q}^{\tiny \mathsf T }&{} 1 \end{array}\right) \end{aligned}$$

where $\textbf{U}$ is a survival matrix and $\textbf{q}$ is a vector of probabilities of mortality. The final row corresponds to an absorbing state. In the data available to us, this absorbing state corresponds to death or loss of health. The block of zeros shows that the dead do not come back to life; the 1 in the lower-right corner is the probability that individuals in the absorbing state remain there.

For example, for 3 age classes the transition matrix is

2 $$\begin{aligned} \textbf{P} = \left( \begin{array}{ccc | c} 0 &{} 0 &{} 0 &{} 0 \\ p_\textrm{H}(1) &{} 0 &{} 0 &{} 0 \\ 0 &{} p_\textrm{H}(2) &{} 0 &{} 0 \\ \hline q_\textrm{H}(1) &{} q_\textrm{H}(2) &{} q_\textrm{H}(3) &{} 1 \end{array}\right) . \end{aligned}$$

Markov chains with rewards were first developed by Howard (1960) in the context of dynamic programming. Caswell (2011) extended the approach to demographic applications with random rewards in the context of lifetime reproduction (van Daalen & Caswell, 2017). Markov chains with rewards were applied in the stochastic analysis of population health by Caswell and Zarulli (2018) for prevalence-based health models and Caswell and van Daalen (2021) for incidence-based models.

The Markov chain with rewards model tracks an individual through their life. At each transition, until reaching the absorbing state, the individual collects a reward, which is a random variable. The model calculates all the moments of the lifetime accumulated reward, from any starting age.

In our case the reward is a year of healthy life. The first two moments of lifetime reward (i.e., the first two moments of healthy longevity) are given by the vectors

3 $$\begin{aligned} \tilde{\boldsymbol{\rho }}_1= & {} \textbf{N}^{\tiny \mathsf T }\textbf{Z} \left( \textbf{P} \circ \textbf{R}_1 \right) ^{\tiny \mathsf T }\boldsymbol{1} \end{aligned}$$

4 $$\begin{aligned} \tilde{\boldsymbol{\rho }}_2= & {} \textbf{N}^{\tiny \mathsf T }\left[ \textbf{Z} \left( \textbf{P}\circ \textbf{R}_2 \right) ^{\tiny \mathsf T }\boldsymbol{1} + 2 \left( \textbf{U} \circ \hat{\textbf{R}}_1 \right) ^{\tiny \mathsf T }\tilde{\boldsymbol{\rho }}_1 \right] , \end{aligned}$$

where $\textbf{P}$ is the transition matrix, $\textbf{N} = \left( \textbf{I} -\textbf{U} \right) ^{-1}$ is the fundamental matrix, and $\textbf{R}_1$ and $\textbf{R}_2$ are the reward matrices containing the first and second moments of the reward associated with each transition among age classes. The symbol ∘ denotes the element-by-element product. The matrix $\textbf{Z}$ cleaves off the absorbing state, $\boldsymbol{1}$ is a vector of ones, and $\hat{\textbf{R}}_k$ is the submatrix of $\textbf{R}_k$ without the absorbing state.

The vectors containing means and variances of remaining health-adjusted years are

5 $$\begin{aligned} E(\hbox {healthy longevity})= & {} \tilde{\boldsymbol{\rho }}_1 \end{aligned}$$

6 $$\begin{aligned} V(\hbox {healthy longevity})= & {} \tilde{\boldsymbol{\rho }}_2 - (\tilde{\boldsymbol{\rho }}_1 \circ \tilde{\boldsymbol{\rho }}_1). \end{aligned}$$

These are vectors whose elements are the remaining healthy life expectancy and variation of remaining healthy life at each age. The mean healthy longevity is exactly the HALE. As a measure of variation, we will focus on the standard deviation of healthy longevity, calculated as the square root of the variance, which we denote as SDHL.

The analysis relies on the age-specific survival probabilities that appear in $\textbf{U}$ and the matrices $\textbf{P}$ and $\textbf{N}$ that are calculated from $\textbf{U}$. It remains only to define the reward matrices $\textbf{R}_1$ and $\textbf{R}_2$. In the calculation of HALE, $q_\textrm{H}(x)$ is the probability of exiting the healthy state, at age *x*, by mortality or by loss of optimal health. The loss could be due to the challenges posed by diseases, disabilities, and/or impairments. The quantity $p_\textrm{H}(x)$ is the complementary probability. Thus, an individual that moves from age class *x* to x+1 remains healthy and accumulates exactly one year of healthy life. An individual that dies or loses health between *x* and x+1 accumulates exactly *a*(*x*) years of healthy life. Such rewards are called ’fixed’ (see van Daalen & Caswell, 2017 for discussion of types of rewards).

The matrix of the first moments of the fixed reward is, for the case of three age classes in Eq. (2),

7 $$\begin{aligned} \textbf{R}_1 = \left( \begin{array}{ccc | c} 0 &{} 0 &{} 0 &{} 0 \\ 1 &{} 0 &{} 0 &{} 0 \\ 0 &{} 1 &{} 0 &{} 0 \\ \hline a(1) &{} a(2) &{} a(3) &{} 0 \end{array}\right) . \end{aligned}$$

Because the rewards are fixed, the second moment matrices are

8 $$\begin{aligned} \textbf{R}_2 = \textbf{R}_1 \circ \textbf{R}_1 \end{aligned}$$

where ∘ denotes the element-by-element product.

## Data

We use data from the Global Burden of Disease Study (Institute for Health, 2019), which has compiled schedules of age-specific HALE for more than 200 countries around the world from 1990 to 2019.^1^ Values are given for ages x=0,1,5 and then by 5-year intervals to age 95.

HALE is as a comprehensive indicator that integrates data on mortality and health outcomes not resulting in death. It denotes the anticipated number of years a person can live in a state of good health, considering both the length and quality of life. The calculation of HALE involves the use of disability weights, which are numerical values assigned to different health states to reflect their impact on overall well-being. These disability weights are applied to the time spent in different health conditions, allowing for the estimation of years lived with disability (YLDs). By combining YLDs with years of life lost (YLLs) due to premature mortality, HALE provides a comprehensive measure of the health status of a population.

Within the framework of HALE, "full health" signifies a condition of well-being where an individual enjoys optimal physical, mental, and social health-a life without the challenges of diseases, disabilities, and impairments. The HALE metric adjusts life expectancy to account for the duration lived in a state less than optimal health.

## Application

The age-specific HALE function implies a life table that generates these values, with the important distinction that the loss of individuals from the initial cohort occurs through either death or the loss of ‘healthy’ status. We call this the ‘health-adjusted life table.’ The probability of being eliminated by either death or loss of health, the health-mortality elimination schedule, we call $q_\textrm{H}(x)$ and was used to apply the matrix model.

To obtain health-adjusted life table from HALE estimates, we used the fact that any column of a life table can be calculated from any other column. The values of HALE provided by GBD can be treated as life expectancy values *e*(*x*) for ages x=0,1,5 and then by 5-year intervals to age 95. We calculated probabilities of death $q_\textrm{H}(x)$ compatible with this *e*(*x*) schedule. We used nonlinear least squares to solve for a $q_\textrm{H}(x)$ schedule that minimizes the squared deviation between our calculated values and the GBD values for *e*(*x*). The resulting $q_\textrm{H}(x)$ schedule was then interpolated to 1-year age intervals with the R-function pclm2D (Pascariu et al., 2018). Age-specific survival probabilities (i.e., probabilities of being alive and in good health at age x+1) are given by $p_\textrm{H}(x)=1-q_\textrm{H}(x)$.

We constrained the final $q_\textrm{H}(x)$ value to be 1.0. Our method also assumes the *a*(*x*) values (the number of person-years lived in the interval by those who exit the population in the interval) are known. This is usually approximated by half of the interval (this means that, on average, the individuals die halfway through the interval). For our estimation, in the absence of specific literature on *a*(*x*) values for a health adjusted life table, we have adopted a conservative approach by assuming that those who leave the healthy state (by death or by acquisition of disability) do so, on average, half-way through the interval. Therefore, we used *a*(*x*) values equal to half of the length of the age interval. The analysis was carried out separately for men and women.

It is important to notice that the definition of healthy longevity in HALE calculations differs in important ways from that of the traditional Sullivan method, or the Markov chain analysis of (Caswell & Zarulli, 2018). In the latter analyses, the age-specific prevalence of disability is used to divide life into healthy and unhealthy at each, implicitly permitting individuals to recover from ill health. The HALE is calculated from the health-adjusted life table, in which the loss of an individual to poor health is as permanent as the loss due to death, so recovery is impossible.^2^

**Fig. 1:**
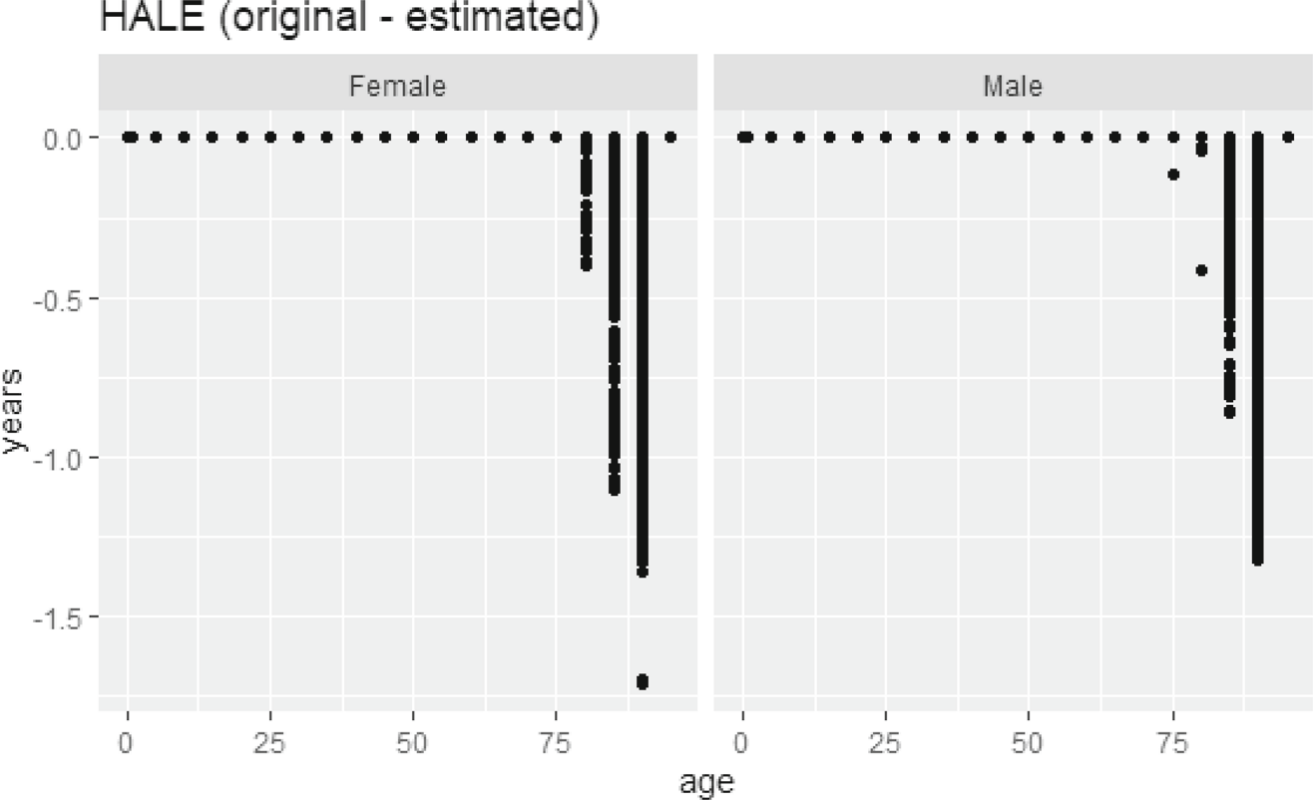
Estimated HALE from authors’ calculation minus HALE reported by Global Burden of Disease (GBD)

A comparison of our estimates of HALE with those reported by GBD revealed an excellent correspondence until age 80 (Fig. 1) Data after age 80 become scanty and the causes of the discrepancy at those ages is an open problem. One plausible explanation is that, after age 80, the combined effects of health deterioration and mortality (referred to as the elimination schedule from the state of full health) occur at values that, on average, differ somewhat from half the width of the interval commonly used in life table calculations for mortality processes alone. Consequently, the rates derived from life table backward calculations after age 80 may not precisely align with the rates used to generate the HALE values in the GBD dataset for those aged 80 and above. This interesting methodological aspect deserved further investigation but is beyond the scope of this analysis.

## Results

From 1990 to 2019, the GBD data shows that HALE at various ages has increased on average in all countries for both men and women. The increment was more substantial at younger ages than at older ages: HALE at birth increased from 59.61 to 64.71 (+5.1 years) for women and from 56.68 to 62.23 for men (+5.55 years), while at age 65 the increase was more modest, from 11.59 to 12.95 (+1.36 years) for women and from 10.01 to 11.39 (+1.38 years) for men. It is important to say that this is the general average trend and that single countries can follow more erratic year-by-year trends.

**Fig. 2:**
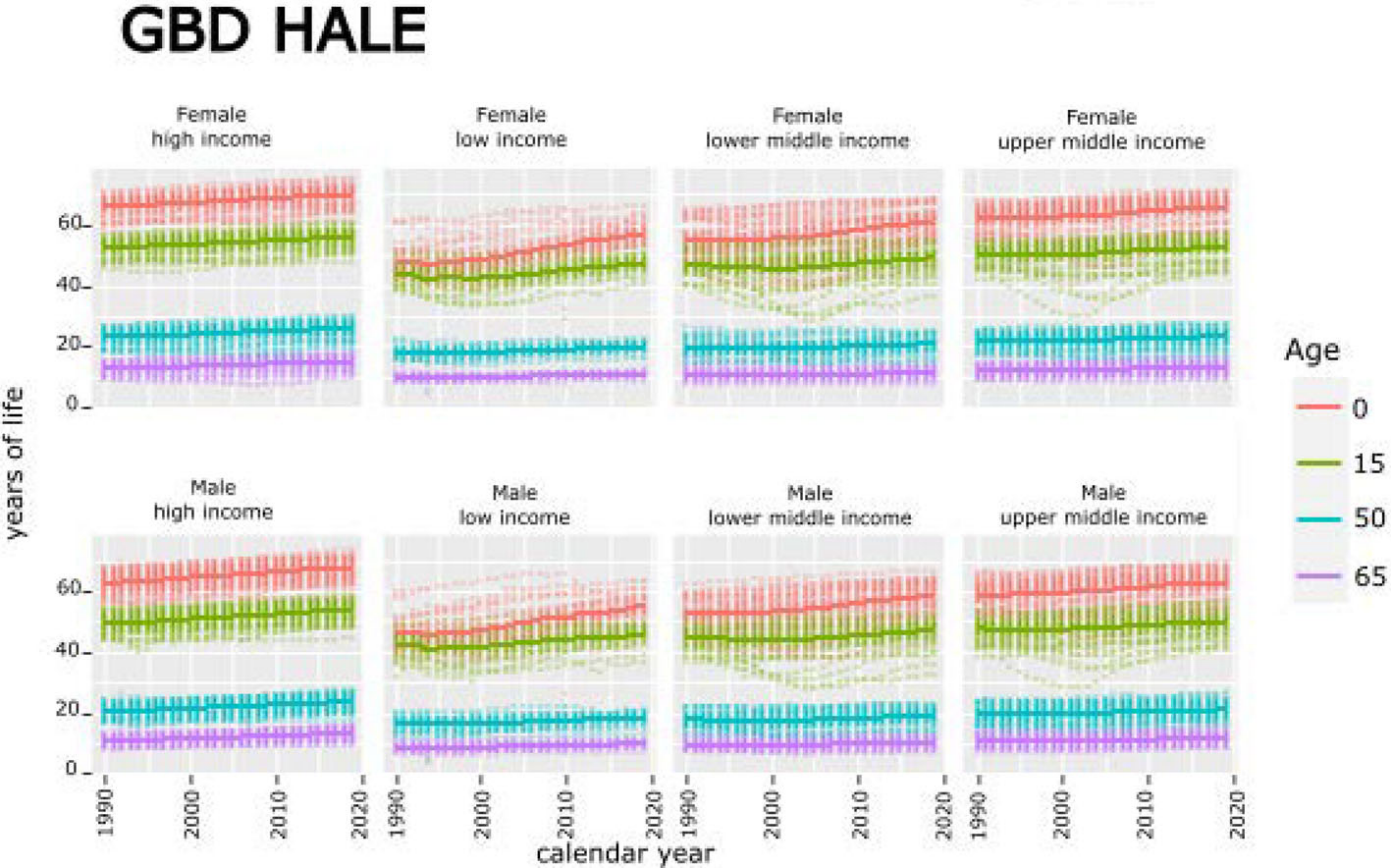
HALE over time at different ages. All countries (faded points) and average value (solid lines). Source: Global Burden of Disease (values from GBD)

The picture is more complex when analyzed according to the income level of the countries (according to the World Bank Income Categorization 2019), as shown in Fig. 2. HALE at birth has increased, on average, more among low-income countries (+9.49 years and +9.48 years for women and men respectively) than among high-income countries (+3.71 years and +4.79 years for women and men respectively): in the low-income countries HALE at birth is now 57.46 years for women and 55.38 years for men, while in the high-income countries it is 69.67 years and 67.45 years for men and women respectively. On the contrary, HALE at older ages has increased more among high income countries than among lower-income countries. For example, HALE at age 50 for women has increased by 2.65 years in high income countries and 1.86 years among low-income countries. The values for men are 3.2 years and 1.93 years respectively. HALE at age 15, instead, has increased more among women in low-income countries than in high income countries (+3.7 years vs. +3.1 years) but more for men in high income countries than in low-income countries (+3.55 vs. +4.06 years).

The analysis of variation among individuals in healthy longevity shows an inverse trend: on average, SDHL at younger ages decreased similarly for both men and women: at age 0 the reduction was of 2.13 years for women and 2.16 years for men and at age 15 it was of 0.74 years and 0.68 years. On the contrary, SDHL at older ages has slightly increased, and slightly more for men than for women: for example, at age 65 the increased was of 0.23 years for men and 0.16 years for women.

**Fig. 3:**
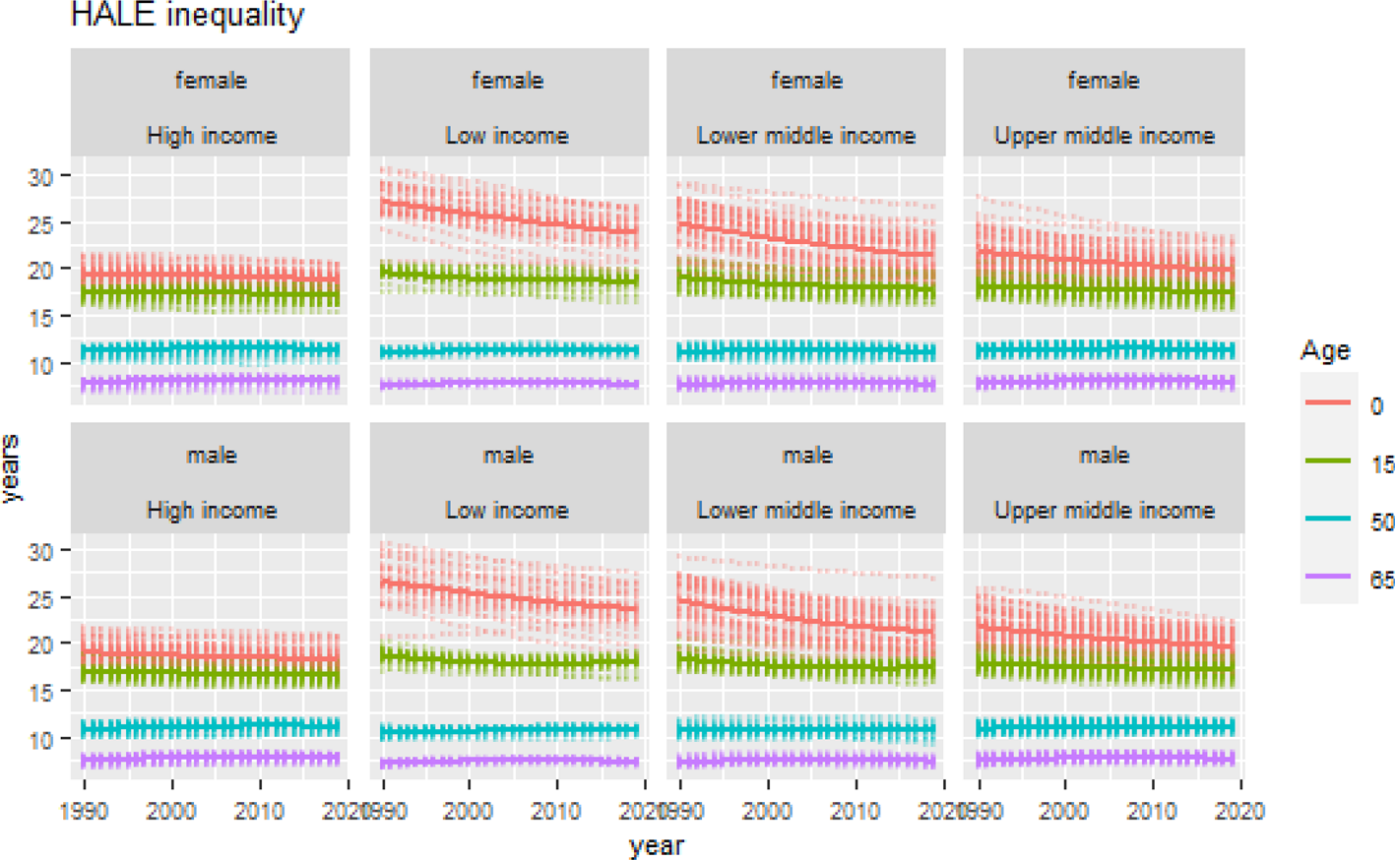
SDHL over time at different ages. All countries (faded points) and average value (solid lines). Source: authors’ calculations

When analyzing the trends by country income groups (Fig. 3), it is striking how the reduction of SDHL at birth (and to a lesser extend also at age 15) has been much more substantial among low-income countries than among high income countries, with 3.5 to 5 times bigger reductions: -3.33 years versus -0.67 years for women and -3.03 years versus -0.88 years for men. At older ages, instead, variation has increased, and it has done so more among high income than among low-income countries, for both men and women: for example, at age 60, in high income countries SDHL grew by 0.23 years for women and by 0.38 for men, while in low-income countries the increment was of 0.16 and 0.19 years for women and men respectively.

**Fig. 4:**
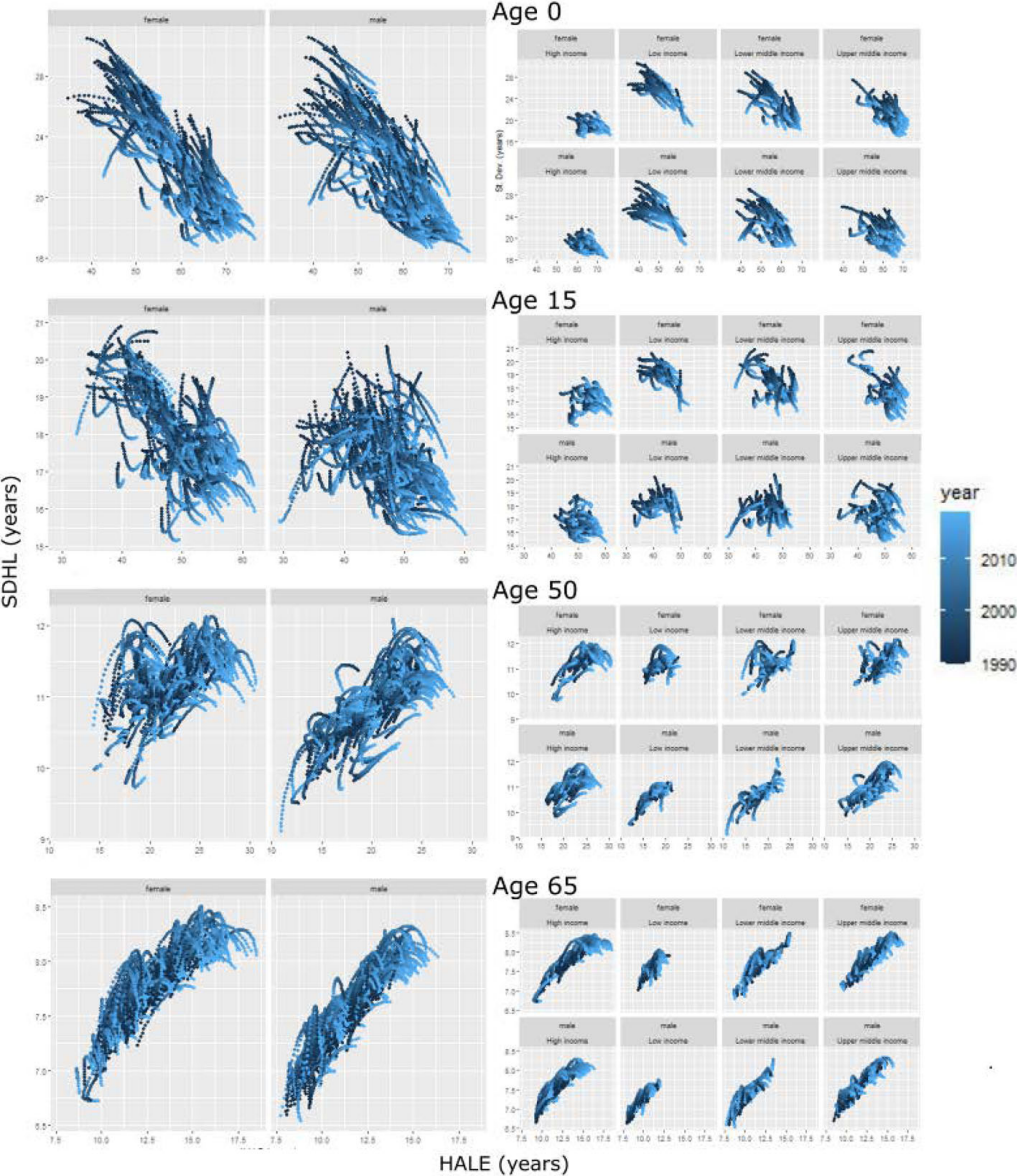
Relation between HALE and STHL at different ages, from 1990 to 2019

Moving beyond the analysis of average trends over time allows us to uncover a much stronger and regular relation, different by age, between healthy lifespan length and variation in healthy lifespan. Figure 4 reports the pairwise relation, for each country in each year, between HALE and SDHL. At birth (and less strongly at age 15), the relation is negative and all the countries, independently of their income level, have decreased the level of variation while increasing healthy life expectancy. The tendency has been more pronounced for low and lower middle-income countries than for upper middle- and high-income countries.

At adult ages the situation changes and the relation between healthy lifespan length and healthy lifespan variation becomes positive. Also, this relation appears valid in all the countries and to be strengthening with age. However, at adult ages the positive relation is more pronounced among upper middle- and high-income countries. Most notably, these countries have increased both the mean and the standard deviation of healthy life span more than the lower middle- and low-income countries.

**Fig. 5:**
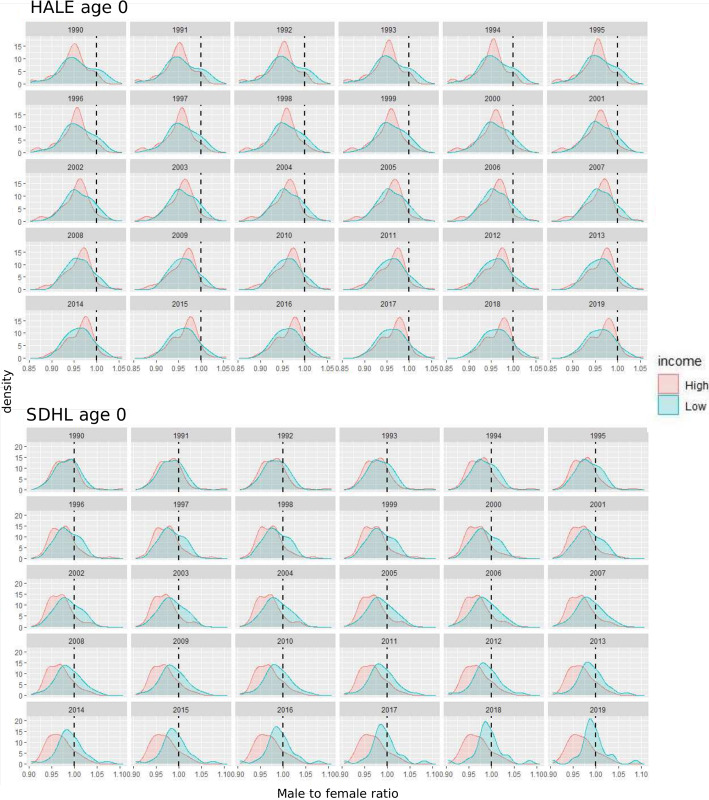
Male to female ratio at birth of HALE (upper panel) and SDHL (lower panel)

**Fig. 6:**
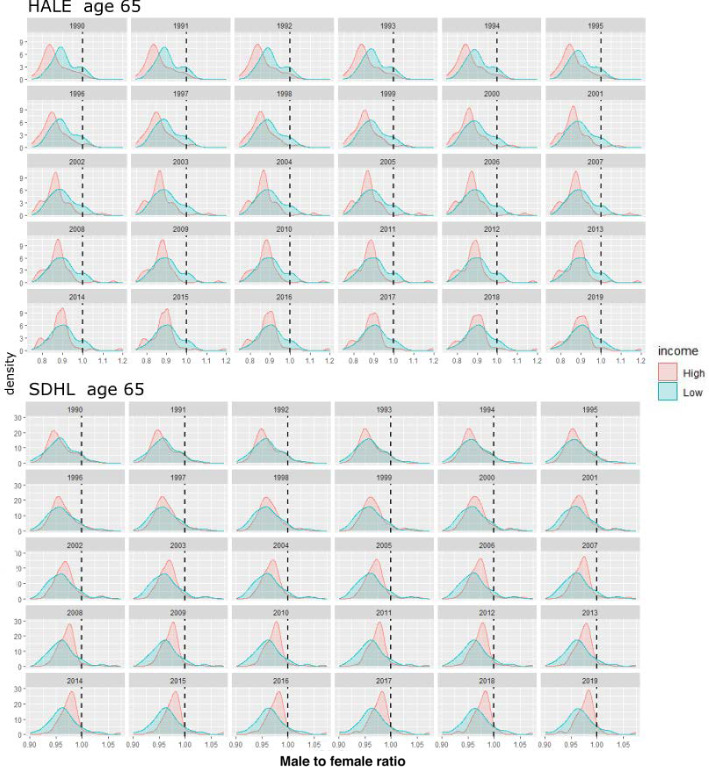
Male to female ratio at age 65 of HALE (upper panel) and SDHL (lower panel)

Women tend to have longer healthy lifespan and higher healthy lifespan variation than men, as indicated by the male to female ratio of both indicators, HALE and SDHL, smaller than 1. Figure 5 shows the density function of these ratios for age 0 and Fig. 6 for age 65. Over time the ratios are consistently below 1 for most of the countries. It is worth noticing that the cases of longer healthy lifespan for men compared to women (and of higher variation) are mostly located in low-income countries compared to high-income ones, both at age 0 and at age 65.

From 1990 to 2019, among high-income countries, male and female healthy life expectancies have become more similar (more at birth than at age 65), as showed by density of the ratio of male to female HALE, that has become more concentrated and whose peak has gradually shifted toward 1 - Figs. 5 and 6, upper panels. A similar trend, but much less pronounced, can be spotted also among low-income countries. Nevertheless, women still benefit from longer healthy life expectancy than men. This difference is particularly pronounced at age 65.

Men and women had, instead, remarkably divergent trends of variation at age 0 in healthy year of life lived among high- and low-income countries - Fig. 5, lower panel. While the ratio in most cases remains solidly below 1, indicating a higher female variation in terms of healthy years of life, the densities for the high- and low- income groups, practically overlapping in 1990, have diverged substantially over time until 2019. At age 0, changes over time have made the two groups of countries diverge: in low-income countries the male to female ratio moved towards 1 and in high-income countries it moved further from 1. The male to female difference in variation of healthy living at age 65, on the contrary, underwent changes over time almost exclusively among high income countries, where the ratio became sharply concentrated around values closer to 1 (Fig. 6, lower panel).

## Discussion

In recent decades healthy life expectancy has increased around the world. Looking beyond expectations to look at variation among individuals is important in aging societies because uncertainty in lifespan is costly, both economically and medically (Edwards, 2013).

Our analysis differs from that of Permanyer et al. (2023) in the analytical methods we employ, based on Markov chains with rewards. This stochastic model offers greater flexibility and analytical power compared to the traditional life table approaches used in other studies. The concept of an individual life represented as a stochastic trajectory along which the individual encounters “rewards” of various kinds is extremely powerful. We have used it here for healthy life extracted from the GBD data, in which death and loss of health both appear as absorbing states. Caswell and Zarulli (2018) have applied it to pure prevalence data and to indices such as DALY (Disability-Adjusted Life Years) that incorporate competing causes of death and disability. It has also been applied to multistate incidence models with almost infinitely diverse set of health measures (Caswell & van Daalen, 2021). Although we have focused on the mean and variance (and standard deviation) here, the Markov chain with rewards method can also provide all the moments of healthy longevity. It can be applied to measures of lifetime fertility (van Daalen & Caswell, 2017), an aspect of health demography that warrants further investigation.

Where our results overlap with those of Permanyer et al. (2023), the similarity is a welcome robustness check using different methods.

We have shown that the relation between healthy lifespan and healthy lifespan variation differs with age. It is negative at young ages and positive ad adult, old ages. As healthy life span at birth and at younger age increased, the variation in healthy lifespan has decreased. As healthy life expectancy at adult and older ages increased, instead, the variation increased. For reduction in variation to happen, it is necessary that the interaction between the age structure of survival and the age-structure of morbidity produces health improvements at ages below specific threshold ages, below which the improvements are translated into a reduction of variation (Aburto et al., 2020; Vaupel et al., 2011).

In contrast, improvements that happen at ages above the threshold ages produce an increase of variation. Our results of a decreasing variation when considering the whole age-range or almost (from birth or from age 15), to increasing variation when focusing only on older ages (from 50 or from 65), indicates that improvements in healthy survival are happening across all the age range. To assess to what extent and with which age-specific differences requires the analysis of the age specific contributions to the relation between healthy life expectancy and healthy lifespan variation. Such analysis goes beyond the scope of this paper, even though it is a relevant and complex topic worth of investigation in a future study.

The most pronounced increase in healthy life expectancy at birth happened among the lower- and lower-middle income countries, which also experienced the sharpest reduction in healthy lifespan variation. Consequently, these countries have reduced the gap with the high- and upper middle- income countries in terms of both average length of healthy life and individual variation around it. This is encouraging and means that from low- to high-income countries, the span of healthy life at birth is getting longer and more and more individuals are benefiting from this prolongment (as indicated by the reduction of the variation).

At older ages, instead, the increase in healthy life expectancy has been more substantial among the high- and upper middle-income countries, as well as the increase of the level of variation. This is a double-sided phenomenon: on the one side, these countries are experiencing a sizeable improvement in healthy survival also at older ages, on the other side, these improvements are more and more unequally distributed among the individuals. Whether this is only due to the formal relation between the improvements in the age-specific healthy survival rates and the dynamics of healthy lifespan-healthy lifespan variation (Aburto et al., 2020; Vaupel et al., 2011), or also to other factors, for example social dynamics, is an important open question awaiting for more research.

The high- low-income countries patterns that we have highlighted partly resemble the phases of the life expectancy revolution (Oeppen & Vaupel, 2002) and the epidemiologic transition (Omram, 2001), which happened first in a group of leading countries, and then gradually spread to the others. The striking leap forward in survival rates and the shift to a disease pattern dominated by degenerative, chronic, and man-made diseases (which tend to affect older ages), have more than doubled the life expectancy and reduced the individual variation and uncertainty around the age at death. The major increases in survival start with dramatic improvements at birth and then gradually shifts to older ages. By the time the forerunners reach the stage where improvements are still possible (almost) only at old ages, the late comers are tackling the initial stage of the phase and experience significant survival improvements at young ages.

The dynamics that we have described so far are not the same for men and women and the gender trends appear different depending on the income level of the country. We have found that women tend to have longer healthy lifespan and higher healthy lifespan variation than men. We also have found that over time women tend to have both a longer mean healthy lifespan and higher variation in healthy lifespan than men, with fewer cases where men have longer healthy lifespan and higher variation compared to women. However, a breakdown of the data by income level of the country reveals, moreover, that these cases are located more in low-income countries than in high-income ones, both at when looking at the indicators of healthy longevity at age 0 and at age 65. Overall, men and women in both groups of countries have experienced an increase of healthy life expectancy, but this was more pronounced among men and in high-income countries. Men and women had, instead, divergent trends of variation. When considered at age 0, in low-income countries, men and women have gradually moved towards similar level of healthy lifespan variation, while in high-income countries they have become more and more different, with women experiencing higher levels of variation than men in most cases. When considered at age 65, sizeable changes (towards more similar level of healthy lifespan variation between the two sexes) were detected only among high-income countries.

## Conclusion

Today people reach older ages in better health than what they used to do only a few decades ago, there here has been a general improvement of healthy survival, a postponement of debility and, overall, healthy life expectancy is increasing. However, to study health improvements it is not enough to look at indicators of average population health, such as healthy life expectancy, or at comparison of healthy life expectancy of population subgroups, such as men versus women. There is a deeper level of inequality that average indicators do not capture: the variation of the distribution of years of health life, that tells us how equally or unequally the healthy life is distributed among the individuals within a population. This is a crucial dimension of populations health because a healthy population is one in which not only people live a long healthy life on average, but also where long healthy lives are enjoyed by as many as possible. To measure progress towards the reduction of health inequalities for as many individuals as possible we need to begin to understand such level of inequality. To do so we need to map and measure the variation of healthy life and analyze its relationship with healthy life expectancy.

In this study we have explored the international landscape of healthy lifespan variation over time. Overall, as the healthy lifespan got longer, its variation decreased but the relation is age dependent. As healthy lifespan increased both at birth and at older ages, the variation decreased at birth but increased at older ages, with important differences: high-income countries showed a more pronounced increase of variation in healthy longevity than lower-income countries and women had higher healthy lifespan variation than men, while they have lower variation than men in total lifespan. In a small, but sizeable, number of cases, men had higher healthy lifespan variation, but this happened predominantly in low-income countries.

The results showed gender specific patterns of healthy lifespan variation, differences by the socio-economic level of the country and, ultimately, that in the realm of healthy longevity, the relation between lifespan length and lifespan inequality might be different compared to what happens in total longevity and it may change with age. Once we scratch the surface and go beyond the sole measurement of healthy life expectancy and start looking and its variation, a complex picture of healthy lifespan-inequality emerges. Such picture calls for future research, to investigate other widely used measures of healthy life and the macro-level factors that influence the observed patterns.

## Footnotes

1 The data are available at https://www.healthdata.org.

2 A full treatment of transitions into and out of good health requires incidence-based analysis based on longitudinal individual data (Caswell & van Daalen, 2021).
